# Herbal medicine and gut microbiota: exploring untapped therapeutic potential in neurodegenerative disease management

**DOI:** 10.1007/s12272-023-01484-9

**Published:** 2024-01-15

**Authors:** Yueyue Guan, Guohua Tang, Lei Li, Jianzhong Shu, Yuhua Zhao, Li Huang, Jun Tang

**Affiliations:** 1 Department of Brain Disease, Chongqing Hospital of Traditional Chinese Medicine, Chongqing, 400021 China; 2 Department of Anorectal Surgery, Hospital of Chengdu University of Traditional Chinese Medicine and Chengdu University of Traditional Chinese Medicine, Chengdu, 610072 China

**Keywords:** Neurodegenerative diseases, Gut microbiota, Herbal medicine, Oxidative stress, Gut–brain axis

## Abstract

The gut microbiota that exists in the human gastrointestinal tract is incredibly important for the maintenance of general health as it contributes to multiple aspects of host physiology. Recent research has revealed a dynamic connection between the gut microbiota and the central nervous system, that can influence neurodegenerative diseases (NDs). Indeed, imbalances in the gut microbiota, or dysbiosis, play a vital role in the pathogenesis and progression of human diseases, particularly NDs. Herbal medicine has been used for centuries to treat human diseases, including NDs. These compounds help to relieve symptoms and delay the progression of NDs by improving intestinal barrier function, reducing neuroinflammation, and modulating neurotransmitter production. Notably, herbal medicine can mitigate the progression of NDs by regulating the gut microbiota. Therefore, an in-depth understanding of the potential mechanisms by which herbal medicine regulates the gut microbiota in the treatment of NDs can help explain the pathogenesis of NDs from a novel perspective and propose novel therapeutic strategies for NDs. In this review, we investigate the potential neuroprotective effects of herbal medicine, focusing on its ability to regulate the gut microbiota and restore homeostasis. We also highlight the challenges and future research priorities of the integration of herbal medicine and modern medicine. As the global population ages, access to this information is becoming increasingly important for developing effective treatments for these diseases.

## Introduction

Neurodegenerative diseases (NDs) are a group of progressive disorders characterized by the degeneration of neuronal structure and function, resulting in cognitive decline and motor dysfunction (Dugger and Dickson 2017). Alzheimer’s disease (AD), Parkinson’s disease (PD), Huntington’s disease (HD), multiple sclerosis (MS), and amyotrophic lateral sclerosis (ALS) are the most common NDs that substantially impact the quality of life of patients (Bloem et al. 2021). The pathogenesis of NDs is intricate and involves multiple factors, such as genetic mutations, environmental influences, and aging (Reitz and Mayeux 2014; Hou et al. 2019). Furthermore, the accumulation of misfolded proteins, neuroinflammation, oxidative stress, and mitochondrial dysfunction are common features of NDs (Lin and Beal 2006). As the aging population increases globally, the prevalence of NDs is increasing, and innovative treatments are needed for disease management.

The human gastrointestinal tract is home to a diverse microbial community, collectively known as the gut microbiota. The gut microbiota is a highly complex microbial community that includes bacteria, viruses, fungi, archaea, and microeukaryotes. Although the more proximal regions of the gastrointestinal tract contain diverse microbial communities, the largest biomass is found in the colon (Guarner and Malagelada 2003). Although most of the current research has focused on bacteria, there is growing evidence that these nonbacterial components may also play important roles in health and disease (Sonnenburg and Sonnenburg 2014). The gut microbiota plays an important role in maintaining overall health by regulating immunity, metabolism, and neurological functions (Milani et al. 2017; Cryan et al. 2019). Emerging evidence suggests that alterations in the composition and function of the gut microbiota are influenced by multiple factors, including host genetics and physiology, environmental exposure, age, and dietary factors, which may contribute to the occurrence and development of NDs (Wu et al. 2011, 2016; Sampson and Mazmanian 2015; Sharon et al. 2016). This link between the gut microbiota and brain function, referred to as the gut–brain axis, highlights the essential role of microbial metabolites, immune modulation, and the vagus nerve in facilitating communication between the gut and the central nervous system (CNS) (Agirman et al. 2021; Mayer et al. 2022). In addition, dysbiosis of the gut microbiota has been linked to increased intestinal permeability, systemic inflammation, and susceptibility to neurodegeneration (Sorboni et al. 2022). Moreover, gut microbiota dysbiosis has been found in patients with AD, PD, HD, and ALS, underscoring the importance of maintaining a balanced gut microbial composition for brain health (Gao et al. 2020).

Herbal medicine has a long history of using herbal compounds to treat various diseases (Liao et al. 2023). Recent studies have shown that certain natural components of herbal medicine can modulate the gut microbiota and exert potential therapeutic effects on NDs (Zhang et al. 2021a). These components, such as berberine, baicalein, and ginsenosides, possess antioxidant, anti-inflammatory, and neuroprotective properties (Fan et al. 2020). In addition, these agents can also modulate the gut microbiota by promoting the growth of beneficial bacteria, inhibiting pathogenic bacteria, and regulating the production of microbial metabolites (Krautkramer et al. 2021). These findings suggest the potential of herbal medicine as an alternative or complementary therapy for NDs.

In this review, we consolidate the current evidence regarding the effects of herbal medicine on the gut microbiota in the context of NDs, with a particular focus on AD, PD, and MS. We reveal the mechanisms by which these herbs modulate the gut–brain axis and their potential implications for preventing and treating NDs. Moreover, we also highlight the challenges and directions for future research.

## NDs and their pathogenesis

**Fig. 1:**
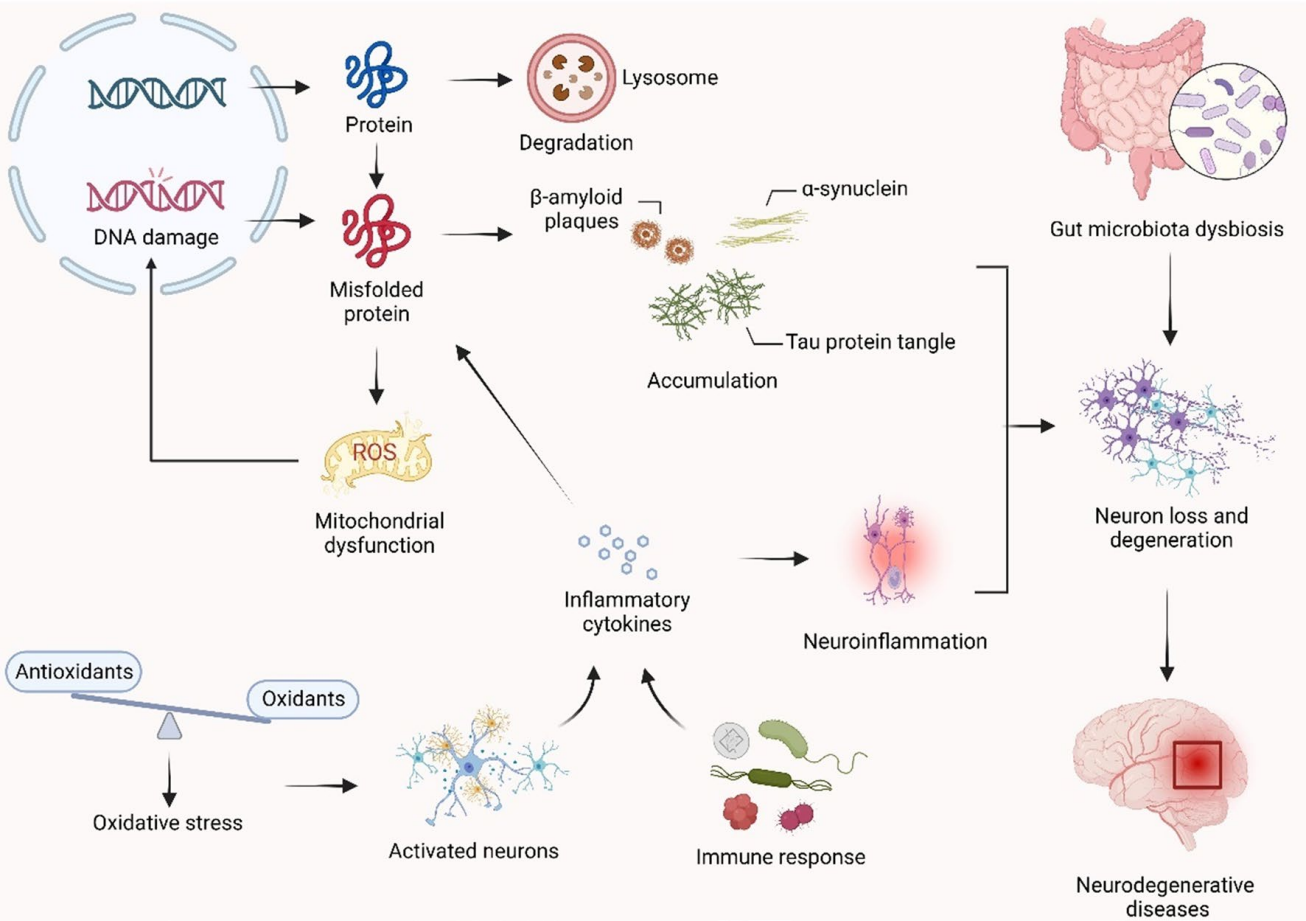
Various factors drive NDs. Under normal homeostatic mechanisms, polypeptides are transcribed and translated from nuclear DNA to extranuclear regions and then properly folded and transported to specific cellular locations. Concurrently, older proteins are labeled and directed to lysosomes for degradation. However, in the event of DNA mutations, translated polypeptides have an increased likelihood of misfolding, resulting in the formation of antidegradation proteins. This leads to the accumulation of misfolded proteins and mitochondrial oxidative stress, driving the pathological progression of NDs. Furthermore, oxidative stress caused by an imbalance between oxidants and antioxidants activates neuronal cells to produce neuroinflammatory factors. These factors synergize with the immune response triggered by pathogen infection, leading to neuroinflammation. Consequently, this inflammation contributes to abnormal biogenesis or protein misfolding, ultimately accelerating the progression of NDs. Additionally, gut microbiota imbalance plays a crucial role in the advancement of NDs

NDs constitute a group of progressive disorders characterized by the loss of neuronal structure and function, resulting in cognitive decline and motor dysfunction. Although the exact pathogenic mechanisms underlying these disorders are incompletely understood, various factors, such as protein misfolding and aggregation, oxidative stress, neuroinflammation, and dysbiosis of the gut microbiota, have been shown to contribute to the development of NDs (Guo et al. 2022) (Fig. 1).

### Protein misfolding and aggregation

Neurons depend on precise protein folding to maintain structural and functional integrity. However, protein misfolding can lead to the formation of toxic protein aggregates, which have been implicated in the pathogenesis of NDs (Soto and Pritzkow 2018). For instance, the accumulation of amyloid-beta (Aβ) plaques and tau tangles serve as hallmarks of AD (Panza et al. 2019). Similarly, PD is characterized by the presence of Lewy bodies containing aggregated α-synuclein (Chen et al. 2022b; Karikari et al. 2022). Furthermore, mutant huntingtin protein aggregation is typical of HD, and TAR DNA-binding protein 43 accumulates in ALS (Highet et al. 2020). These toxic aggregates impair the function of neuronal cells and accelerate neuronal cell death. In addition, recent studies have demonstrated that molecular chaperones and the ubiquitin-proteasome system (UPS) play critical roles in maintaining protein homeostasis and preventing protein misfolding and aggregation (Balchin et al. 2016; Eldeeb et al. 2022). Deficiencies in these systems may result in the accumulation of toxic protein aggregates, causing cellular dysfunction and neuronal death.

### Oxidative stress

Oxidative stress, which refers to an imbalance between reactive oxygen species (ROS) production and antioxidant defense mechanisms, is widely considered to be a vital factor in the progression of NDs (Teleanu et al. 2022; Tesco and Lomoio 2022). Elevated ROS levels can lead to cellular damage, including lipid, protein, and nucleic acid damage, ultimately leading to neuronal dysfunction and death (Ashleigh et al. 2023). In addition, mitochondrial dysfunction, impaired energy metabolism, and increased ROS production are common features of NDs (Elfawy and Das 2019). Notably, both endogenous and exogenous antioxidants can help neutralize ROS and mitigate oxidative stress, providing a potential therapeutic avenue for these diseases (Kabir et al. 2022). Interestingly, current promising therapeutic strategies for counteracting oxidative stress involve the use of antioxidants and targeting cellular antioxidant mechanisms, such as the nuclear factor erythroid 2-related factor 2 (Nrf2) pathway and its downstream targets, to protect against neurodegeneration (Jazvinšćak Jembrek et al. 2021; Ulasov et al. 2022).

### Neuroinflammation

Neuroinflammation is a prominent feature of NDs and contributes to neuronal death. For instance, microglia are the primary immune cells in the CNS and play a crucial role in the regulation of neuroinflammation by releasing proinflammatory cytokines such as tumor necrosis factor-alpha (TNF-α), interleukin-1β (IL-1β), and IL-6 (Xu et al. 2020; Woodburn et al. 2021). Notably, recent research has revealed the key role of inflammasomes, such as NLRP3, in microglial activation and the development of NDs (Jin et al. 2019; Ahmed et al. 2021). Therefore, targeting neuroinflammation by modulating microglial activation, proinflammatory cytokine production, or inflammasome signaling is a promising therapeutic approach for NDs.

### Gut microbiota dysbiosis

Gut microbiota dysbiosis also plays an essential role in the pathogenesis of NDs. There is growing evidence suggesting that alterations in the composition of the gut microbiota can contribute to NDs by affecting neurotransmitter production, and modulating neuroinflammation, immune responses, oxidative stress, protein aggregation, and gut microbiota dysbiosis (Liu et al. 2020b). Furthermore, recent studies have shown the potential benefit of targeting the gut microbiota through dietary interventions, probiotics, and fecal flora transplantation to treat NDs (Peng et al. 2020).

In conclusion, understanding the complex relationships between protein misfolding and aggregation, oxidative stress, neuroinflammation, and gut microbiota dysbiosis in the pathogenesis of NDs is crucial for the development of novel therapeutic approaches targeting these pathways. Recent advances in these areas have shed new light on the development of potential therapeutic targets and strategies for NDs.

### Gut microbiota and NDs

**Fig. 2:**
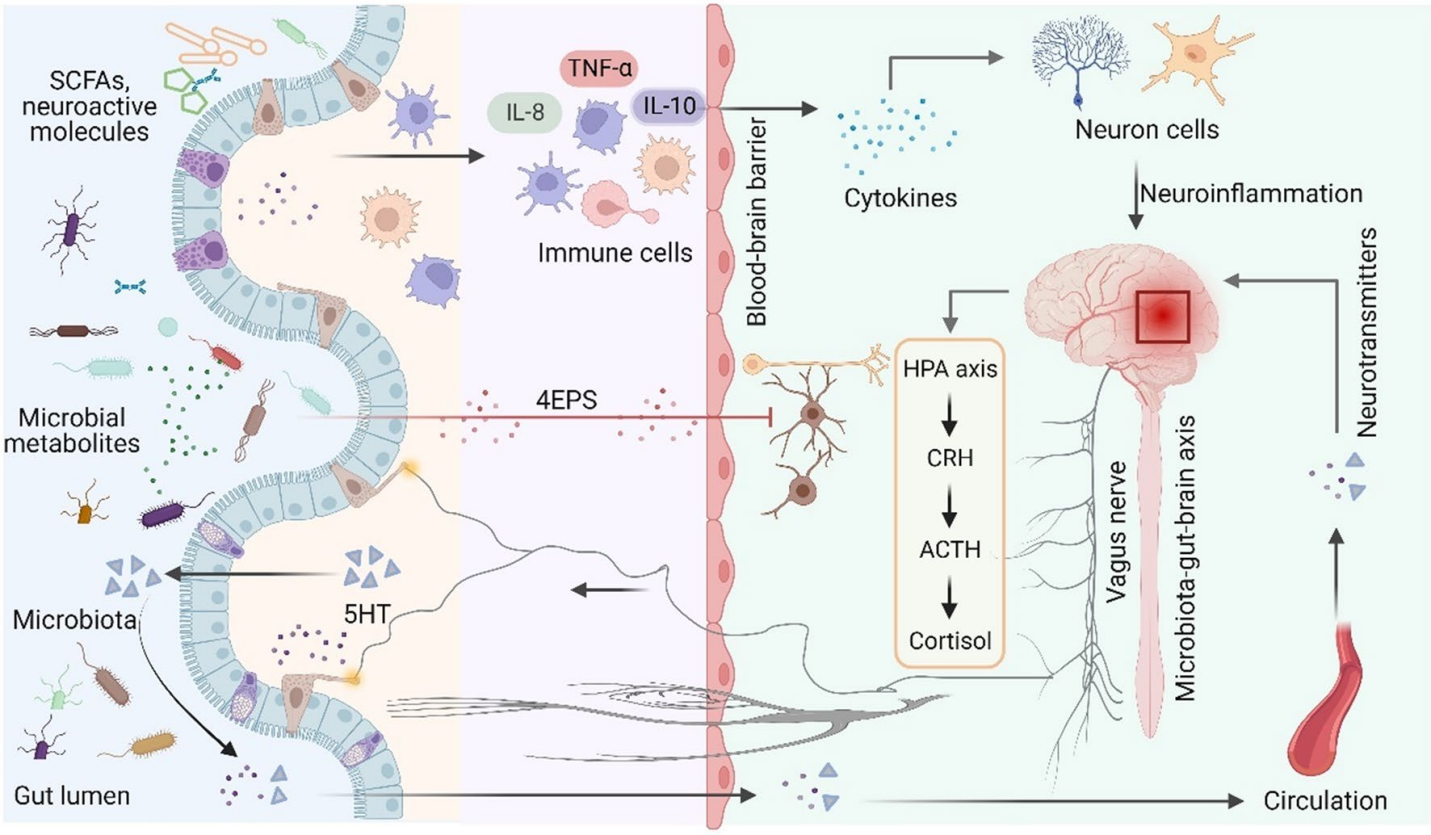
Microbiota-gut-brain axis**.** The brain and gut are closely linked through neural, metabolic, endocrine, and immune pathways. The brain regulates intestinal health through the vagus nerve, the hypothalamic‒pituitary‒adrenal (HPA) axis, and systemic circulation. At the same time, the intestinal microbiota can regulate the intestinal barrier and nerve cell function through the bacteria itself, microbial-derived metabolites (such as 5-HT and 4EPS), peripheral immune cells, and their secretory factors, thus affecting brain function

The gut–brain axis plays a vital role in maintaining overall health (Fig. 2). This intricate network involves multiple nervous and immune systems and is influenced by the composition of the gut microbiota and its metabolites (Mayer et al. 2022). Notably, the gut–brain axis contributes to regulating various physiological processes, including digestion, metabolism, the immune response, and mood (D’Antongiovanni et al. 2023). Multiple communications of the gut–brain axis occur through several pathways, such as neural connections, hormonal signaling, immune interactions, and microbial metabolites (Ancona et al. 2021). The enteric nervous system, often referred to as the “second brain, ” is a complex network of neurons embedded in the gastrointestinal tract that communicate with the central nervous system (CNS) through the autonomic nervous system (ANS) and the hypothalamic-pituitary-adrenal axis (HPA) (Furness 2012). Notably, the gut microbiota plays a vital role in this communication by producing neurotransmitters and modulating host metabolism (Strandwitz 2018). Emerging evidence suggests that disturbances in the gut-brain axis contribute to the progression of multiple diseases, including irritable bowel syndrome (IBS), inflammatory bowel disease (IBD), and other mental disorders, such as anxiety and depression (Raskov et al. 2016). Therefore, understanding the mechanisms underlying the gut–brain axis and interactions with the gut microbiota has become a vital area of research for ensuring overall health and well-being.

The relationship between the gut microbiota and NDs has garnered substantial attention in recent years (Table 1). Accumulating evidence has revealed the vital role of the gut microbiota in the onset and progression of neurological disorders by modulating brain function and behavior (Ma et al. 2019). Alterations in the gut microbiota are associated with several NDs, including AD, PD, HD, and MS. For instance, dysbiosis of the gut microbiota is associated with the production of amyloid‒β plaques and neuroinflammation, both of which are critical factors in the pathogenesis of AD (Shabbir et al. 2021). In addition, certain gut microbial metabolites, such as short-chain fatty acids (SCFAs) and tryptophan derivatives, have shown neuroprotective effects by modulating the immune response of the CNS and enhancing the integrity of the blood-brain barrier (Wang et al. 2023b). In addition, alterations in the gut microbiota have also been reported, with an increase in proinflammatory bacterial species and a decrease in SCFA-producing bacteria in PD patients (Martin-Gallausiaux et al. 2021). The role of the gut microbiota in the pathogenesis of PD is further supported by the observation that α-syn aggregation is a hallmark of PD (Rutsch et al. 2020). Similarly, MS has been linked to alterations in the composition of the gut microbiota, characterized by a decrease in anti-inflammatory and neuroprotective bacterial species. These alterations can affect the blood-brain barrier and promote neuroinflammation, which accelerates the development of MS (Jangi et al. 2016). In conclusion, a deeper understanding of the interactions between the gut microbiota and the gut–brain axis is essential for elucidating the mechanisms of NDs.

**Table 1:** Involvement of gut microbiota in NDs

Bacterial genus	Mechanisms	Experimental Subject	Related NDs	References
*Escherichia*, *Staphylococcus*, *Bacillus*, *Klebsiella*, *Salmonella* *Lactobacillus*	Bacterial amyloid, cortical excitability and neural excitation-inhibition	Male patients with AD; Tg2576 mice	AD	(Cao and Mezzenga 2019; Ciminelli et al. 2020)
*Bifidobacterium*	GABA, cortical excitability, and neural excitation-inhibition	Fecal samples from AD	AD	(Auger et al. 2020)
*Bacteroides*	Change of cerebral amyloid-β plaques and neurofibrillary tangles	Male patients with AD	AD	(Chen et al. 2022a)
*Roseburia*, *Faecalibacterium*	Produce SCFAs	16 S microbiome datasets	PD	(Nuzum et al. 2020)
*Pseudomonas*	Fap, change of α-synuclein	Patients with PD	PD	(Christensen et al. 2019)
*Enterobacteriaceae*	Curli, α-synuclein aggregation	Patients with PD	PD	(Sampson et al. 2020)
Hydrogen-product bacteria	Reduced dopaminergic loss	/	PD	(Fujita et al. 2009)
*Coriobacteriales*, *Erysipelotrichales*, *Bacteroidales*, *Burkholderiale*	Unknown	Male patients with HD	HD	(Radulescu et al. 2020)
*Eubacterium* *hallii*	Unknown	Amyloid-positive patients	HD	(Love et al. 2022)
*Acidaminococcaceae*, *Bacteroidaceae*, *Lachnospiraceae*	Functional pathways and enzymes	Fecal samples	HD	(Wasser et al. 2020)
*Clostridium*	Decrease level of SCFA secretion	Patients	MS	(Miyake et al. 2015)
*Lactococcus* *lactis*, *Bifidobacterium* *lactis*	Anti-inflammatory	16 S microbiome datasets	MS	(Riccio and Rossano 2018)
*Firmicutes*, *Bacteroidetes*, *Prevotella*	Unknown	Fecal samples; Symptomatic Tg2576 mice	MS	(Chen et al. 2016)

### Herbal medicine and NDs

Traditional Chinese medicine (TCM) has a history spanning thousands of years and constitutes an essential element of Chinese culture and health care. Central to TCM is the use of herbal medicine, which has played a significant role in preventing and treating various diseases throughout China’s long history (Zhang et al. 2021c). Notably, with its holistic approach, TCM not only focuses on symptoms but also emphasizes balance and harmony among the individual’s body, mind, and environment (Chan et al. 2012). Herbal medicine encompasses thousands of different medicinal plants, minerals, and animal-derived substances. For instance, ancient texts, such as Huangdi Neijing (Yellow Emperor’s Inner Canon) and Shennong Bencaojing (Classic of Herbal Medicine), have laid the foundation for modern TCM, documenting numerous herbs and their therapeutic properties (Zhang et al. 2022b). In recent years, Western scientific research has confirmed the efficacy of herbal medicine, leading to increased global interest in its potential health benefits.

### Therapeutic effects of herbal medicine on NDs

The therapeutic principles of herbal medicine are rooted in the ancient TCM concepts of yin and yang, qi (vital energy), and five elements (wood, fire, earth, metal, and water). These principles guide the diagnosis and treatment of diseases by addressing imbalances and deficiencies in the body’s vital energy, blood circulation, and organ functions (Fu et al. 2021; Pun and Chor 2022). Notably, herbal treatment in TCM aims to restore and maintain harmony within the body by addressing the underlying causes of disease rather than just alleviating symptoms. Doctors often use personalized prescriptions that involve the combination of multiple herbs in specific proportions to address the patient’s unique condition and imbalance. Interestingly, this synergistic approach allows for the enhancement of therapeutic effects while minimizing adverse side effects. In TCM, herbs are categorized based on their flavor, temperature, and therapeutic actions, which determine their effects on specific organs and body functions. The therapeutic goals of herbal medicine include strengthening the immune system, promoting blood circulation, eliminating toxins and pathogens, and regulating organ function.

Recently, herbal medicine has gained attention for its potential role in treating NDs. Numerous herbs and formulations have shown promise in clinical trials (Zhu et al. 2022; Liu et al. 2023a) (Table 2). For instance, several herbs have shown potential benefits in alleviating cognitive decline and addressing the pathological hallmarks of AD. These herbs include *Huperzia serrata*, from which the natural product huperzine A is isolated and it demonstrates acetylcholinesterase inhibition and a reduction in β-amyloid plaques (Friedli and Inestrosa 2021). *Ginkgo biloba* contains a variety of bioactive components, such as polysaccharides, flavonoids, terpenoid trilactones, and ginkgolic acids, which are known for their antioxidant and anti-inflammatory properties. *Polygala tenuifolia* contains the main bioactive ingredient senegenin and exhibits neuroprotective effects (Martínez-Solís et al. 2019; Wang et al. 2022a). A commonly used formula for cognitive disorders is Liuwei Dihuang Wan, which aims to nourish yin and strengthen the kidneys, thereby improving memory and cognitive function (Wu et al. 2007). In addition, herbal treatments often focus on replenishing depleted neurotransmitters and protecting dopaminergic neurons in PD patients. Notable herbs, including *Uncaria rhynchophylla* (Gou Teng with major components, such as rhynchophylline and isorhynchophylline), with its neuroprotective properties (Wang et al. 2018; Yang et al. 2020), and *Cistanche deserticola* (Rou Cong Rong with total glycosides as the main active components), help improve dopamine levels (Li et al. 2016; Wang et al. 2020). Notably, a frequently prescribed formula for PD is Zhenwu Tang, which warms and tonifies yang while expelling dampness. Herbal medicine focuses on reducing inflammation and enhancing immune function. *Scutellaria baicalensis* (Huang Qin), which contains the main bioactive ingredients baicalin (BA) and baicalein (BE), is known for its anti-inflammatory and neuroprotective effects, while *Astragalus membranaceus* (Huang Qi) boosts the immune system in MS (Zhao et al. 2019). Furthermore, a commonly used formula for MS treatment is Bu Zhong Yi Qi Tang, which aims to tonify qi and strengthen the spleen (He et al. 2017). In conclusion, herbal medicine shows promising potential in the prevention and treatment of NDs. More in-depth research and related clinical trials are needed to verify the efficacy and safety of these herbs and formulations and to clarify their potential mechanisms of action.

**Table 2:** The effect and mechanism of the representative herbal medicine in NDs

Herbal medicine	Sources	Mechanisms	Related NDs	References
Quercetin	Apples, berries, onions, and capers	Inhibition of misfolded proteins, anti-inflammatory, antioxidation	AD, PD, HD, ALS	(Chakraborty et al. 2015; Ip et al. 2017)
Resveratrol	Grapes, raspberries, mulberries, and peanuts	Inhibition of misfolded proteins, anti-inflammatory, antioxidation	AD, PD, HD, ALS	(Mancuso et al. 2014)
Schisandrin B	*Schisandra* *chinensis*	Inhibition of misfolded proteins, anti-inflammatory, antioxidation	AD, PD, HD	(Lam and Ko 2012; Zhang et al. 2017)
Tea leaves	Tea	Inhibition of misfolded proteins, anti-inflammatory, antioxidation, anti-apoptosis	AD, PD	(Chen et al. 2015; Li et al. 2019)
*Scutellaria* *baicalensis*	The root of *Scutellaria baicalensis* Georgi	Inhibition of misfolded proteins, anti-inflammatory, antioxidation, anti-apoptosis	AD, PD	(Li et al. 2005; Sonawane et al. 2019)
*Lycium* *barbarum*	*L*.* barbarum* L. fruit	Inhibition of misfolded proteins, anti-inflammatory, antioxidation, anti-apoptosis	AD	(Zhang et al. 2013)
*Schisandra* *chinensis*	The dry ripe fruit of *Schisandra chinensis*	Antioxidation, regulation of neurotransmitters, anti-inflammatory, anti-apoptosis	AD, PD, HD	(Zhang et al. 2018)
Nobiletin	Citrus fruits	Decreasing the expression of HMGB-1 and pyroptosis-related proteins	AD	(Chai et al. 2022)
Salidroside	The tubers of *Rhodiola* plants	Inhibiting NLRP3 inflammasome-mediated pyroptosis	AD, PD	(Zhang et al. 2020; Cai et al. 2021)
*Lonicera* *japonica* Thunb	/	Mitochondrial enzyme complex activity, mitochondrial function	PD	(Sun et al. 2005)
Curcumin	Turmeric	Antioxidation, autophagy, anti-inflammatory	AD	(Li et al. 2011; Gong and Sun 2022)
Berberine	Chinese herb Coptis chinensis and other Berberis plants	Neuroprotection, antioxidation, anti-apoptosis	AD	(Chen et al. 2020; Li et al. 2021a)
Triptolide	Tripterygium wilfordii Hook	Anti-apoptosis	AD	(Xu et al. 2016)
Vitegnoside	Medicinal plant Vitex negundo	Anti-inflammatory, antioxidation, anti-apoptosis	AD	(Wang et al. 2019)

### Mechanisms underlying the effects of herbal medicine in the treatment of NDs

**Fig. 3:**
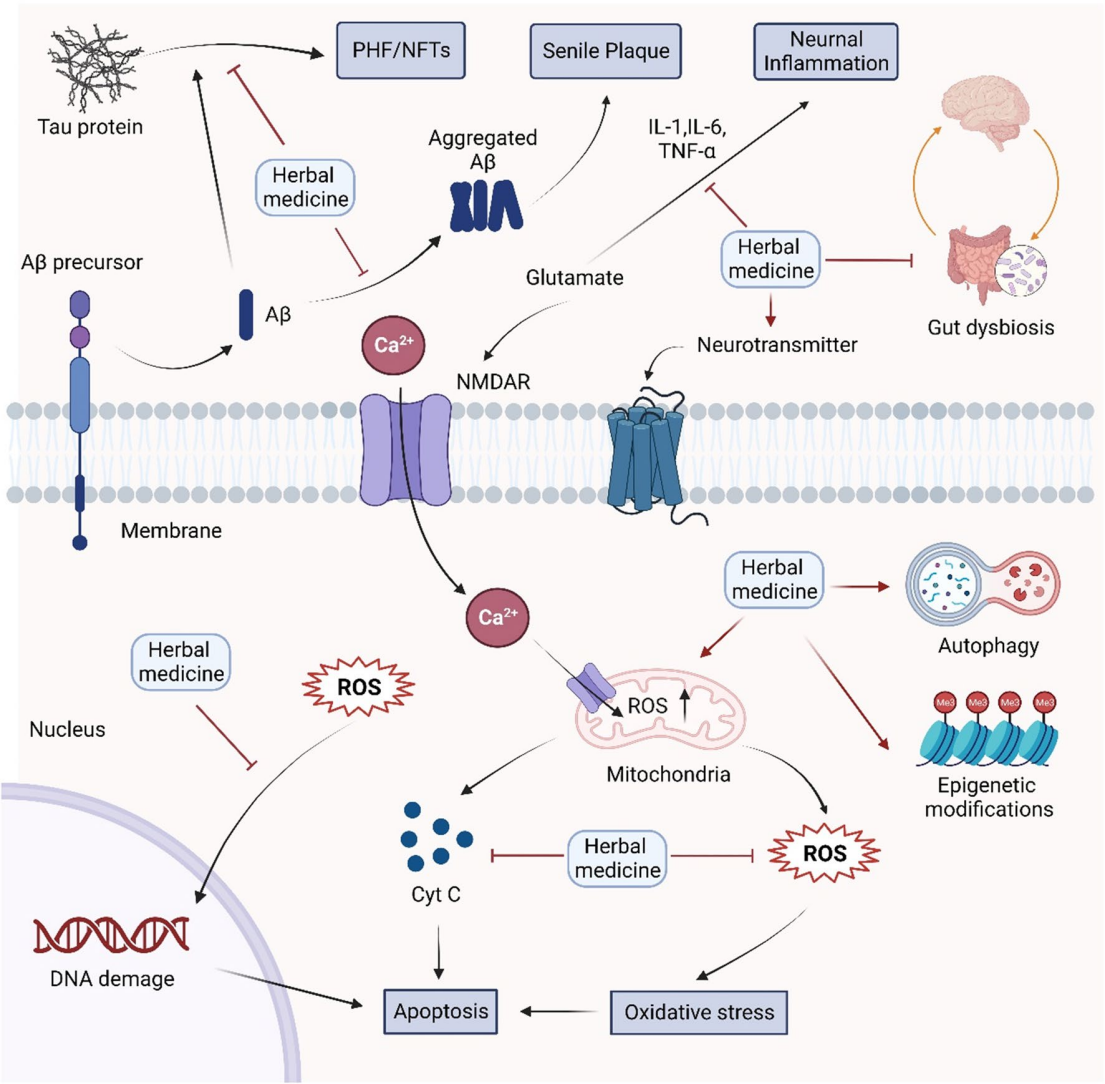
Mechanisms underlying the effects of herbal medicine on NDs. Herbal medicine has the potential to delay the accumulation of amyloid (amyloid β-protein) Aβ protein and Tau protein, regulate the release of central cholinergic and other neurotransmitters, and protect neurons against apoptosis and oxidative stress, thereby providing neuroprotection. Moreover, herbal medicine can also modulate autophagy, enhance mitochondrial function and biogenesis, regulate epigenetic modifications, and influence the gut-brain axis through its interactions with gut microbiota. These multifaceted effects contribute to the prevention and mitigation of NDs

Herbal medicine has been widely used for thousands of years to treat various diseases, especially NDs. It is necessary to reveal the molecular mechanism of TCM treatment of NDs, emphasizing its antioxidant, anti-inflammatory, antiapoptotic, and neuroprotective properties, among others (Fig. 3).

### Antioxidative and anti-inflammatory actions

Oxidative stress plays a vital role in the pathogenesis of NDs through protein oxidation, lipid peroxidation, and DNA damage (Esmaeili et al. 2022). Neuroinflammation is closely associated with NDs and exacerbates neuronal dysfunction and death (Scuderi et al. 2020). Herbal medicines can exhibit anti-inflammatory effects through multiple mechanisms, including the inhibition of proinflammatory cytokines, the suppression of microglial activation, and the blockade of inflammatory signaling pathways, thus demonstrating utility in the treatment of NDs (Gong et al. 2020a; Liu et al. 2022b; Li et al. 2023a).

### Antiapoptotic and neuroprotective effects

Apoptosis, or programmed cell death, is associated with the pathogenesis of NDs caused by neuronal loss. Herbal medicines can exert antiapoptotic effects by modulating key regulators of apoptosis, including Bcl-2 family proteins, caspases, and p53. For instance, by enhancing the expression of antiapoptotic proteins (e.g., Bcl-2 and Bcl-xL) and reducing the levels of proapoptotic proteins (e.g., Bax and Bad), herbal medicine can restore the balance between cell survival and death, ultimately protecting neurons from degeneration (Liu et al. 2021a).

Herbal medicine provides neuroprotection through a variety of mechanisms, such as promoting neurogenesis, enhancing synaptic plasticity, and regulating the neurotransmitter system (Liu et al. 2021b). For instance, herbal medicine can stimulate neural stem cells to proliferate and differentiate into functional neurons by modulating related signaling pathways such as the Wnt/β-catenin and Notch pathways (Zhou et al. 2014; Li et al. 2023c). Notably, herbal medicine can improve learning and memory functions by facilitating long-term potentiation and modulating neurotransmitter levels, such as glutamate, dopamine, and acetylcholine (Wu et al. 2019; Liu et al. 2020c).

### Modulation of protein misfolding and aggregation

Recent research has revealed that herbal medicines can modulate protein misfolding and aggregation, which are hallmarks of several NDs. In addition, certain components of herbal medicine can prevent Aβaggregation, promote Aβ clearance, and inhibit tau hyperphosphorylation in AD (Lu et al. 2021).

### Herbal medicine metabolism

In the context of TCM, the gut microbiota acts as an “unseen pharmacist” capable of biotransforming herbal constituents into active metabolites, thereby affecting their bioavailability and pharmacological activity. Ginseng, one of the most widely used herbs in TCM, contains a plethora of saponins known as ginsenosides (Mancuso and Santangelo 2017). While these ginsenosides have numerous reported health benefits, some of their pharmacological actions are attributed to their metabolic products rather than the parent compounds. A key player in this metabolic transformation is compound K, a secondary metabolite formed through the action of intestinal bacteria on primary ginsenosides such as Rb1 and Rb2. Compound K is particularly noteworthy because it has been shown to exert significant anti-inflammatory, antioxidative, and anticancer effects (Choi and Kim 2023; Liu et al. 2023b; Tian et al. 2023). These effects are often more potent than those of the original ginsenosides, highlighting the importance of gut microbial metabolism in enhancing the pharmacological efficacy of ginseng.

Research into the interactions between herbal medicine components and gut microbiota is still evolving. Nevertheless, several studies have underscored the necessity of considering the microbiome when evaluating the pharmacokinetics and pharmacodynamics of herbal medicines. In conclusion, the gut microbiota serves as a critical mediator of the pharmacological effects of herbal medicines, such as ginseng. By transforming inert compounds into bioactive metabolites, these microscopic inhabitants of the human gut significantly influence the therapeutic outcomes of herbal medicine.

### Other mechanisms

In addition to the mechanisms discussed above, herbal medicine may also impact other cellular processes relevant to NDs. These processes include the modulation of autophagy, enhancement of mitochondrial function and biogenesis, regulation of epigenetic modifications, and modulation of the gut–brain axis through interaction with the gut microbiota (Zhu et al. 2020a; Wang et al. 2021b).

In conclusion, herbal medicine holds great promise for treating NDs due to its pleiotropic effects on multiple molecular pathways. Further research is warranted to determine the detailed mechanisms underlying the therapeutic effects of herbal medicine and develop effective treatment strategies that can be integrated into mainstream medicine.

### The impact of herbal medicine on the gut microbiota

**Fig. 4:**
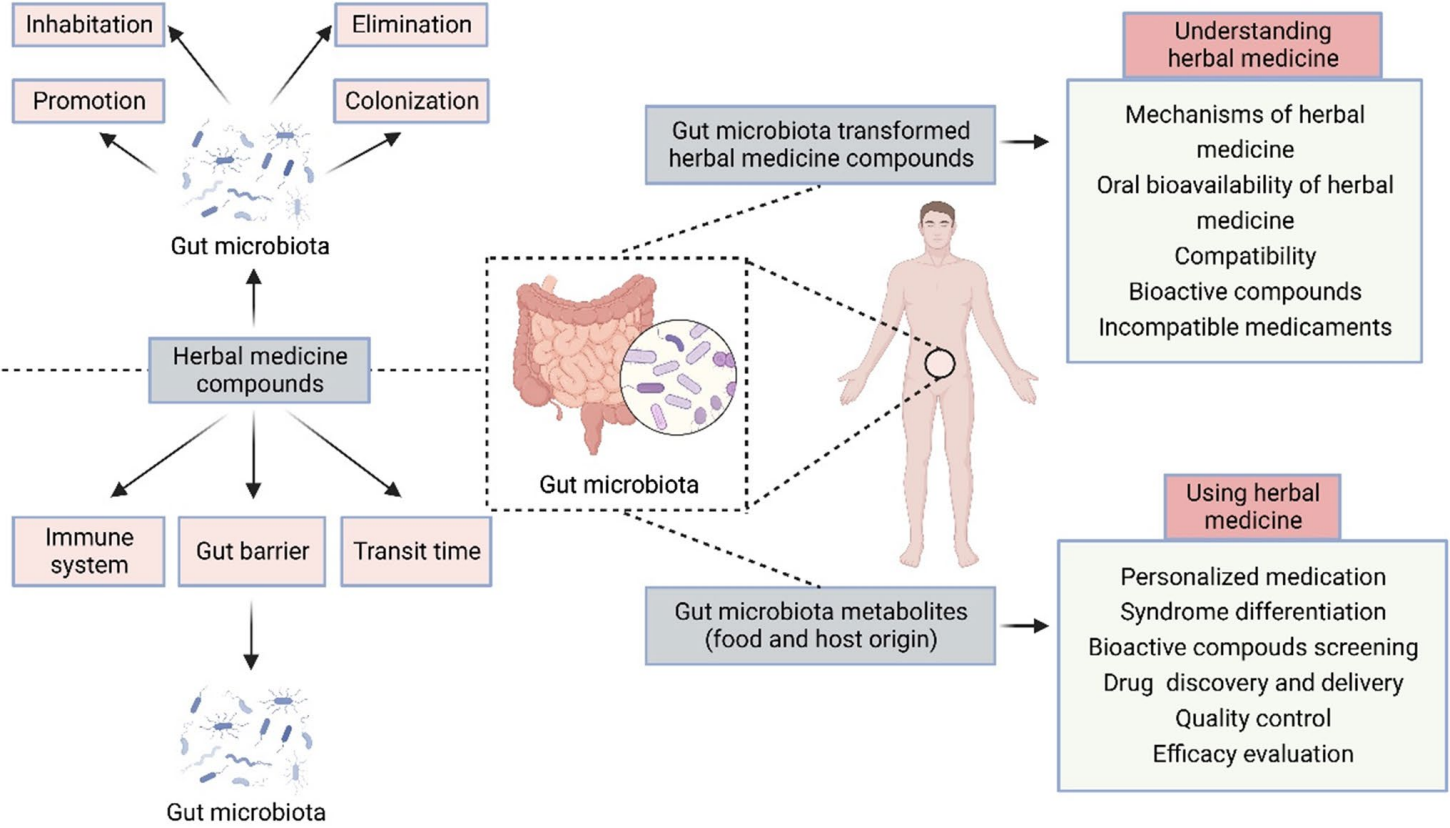
The effects of herbal medicine on gut microbiota composition. The effects of herbal medicine on gut microbiota were classified as inhabitation, promotion, elimination, and colonization. In addition, the regulatory effects of herbal medicine on gut microbiota composition also include enhancing gut barrier function, inducing host immune response, regulating the composition and metabolism of gut microbiota, changing transport time, etc. More importantly, gut microbiota can enhance our understanding and use of herbal medicine in many ways, which provides more possibilities for herbal medicine to treat diseases

Recent research has revealed the considerable impact of the gut microbiota on multiple aspects of human health, ranging from digestion and metabolism to immunity and mental well-being (Clemente et al. 2012; Fan and Pedersen 2021). Herbal medicine has been shown to influence the composition and function of the gut microbiota. This influence plays a vital role in the therapeutic effects of herbal medicine (Xu et al. 2017) (Fig. 4). Furthermore, recent research has shown that herbal medicine can modulate the composition of the gut microbiota, resulting in increased diversity and favorable shifts in microbial communities. These changes often correlate with improved health outcomes, such as reduced inflammation, enhanced immune responses, and better nutrient absorption (Gong et al. 2020b; Xia et al. 2022a). Moreover, the use of herbal medicine for treating metabolic diseases, such as obesity and type 2 diabetes mellitus, has also been associated with alterations in the composition and function of the gut microbiota (Zhang et al. 2021b). For instance, several mechanisms underlie the effects of herbal medicine on the gut microbiota, including prebiotic effects, antibacterial properties, and immunomodulation.

### Prebiotic effects

Some Chinese herbs contain nondigestible polysaccharides, oligosaccharides, and other bioactive compounds that serve as substrates for specific bacterial species in the gut. These components act as prebiotics, promoting the growth and activity of beneficial bacteria while inhibiting pathogenic bacteria (Liu et al. 2022c). For instance, *Astragalus membranaceus* (Huang Qi) and *Lycium barbarum* (Goji berries) are rich in polysaccharides that specifically stimulate the growth of *Bifidobacterium* and *Lactobacillus* (Zhu et al. 2020b; Ding et al. 2021). These prebiotic effects can improve the overall gut microbial composition, enhance SCFA production, and maintain gut barrier integrity, ultimately contributing to improved gut health and disease prevention.

### Antibacterial properties

Numerous components of herbal medicine exhibit direct antibacterial effects that can suppress the growth of harmful bacteria while promoting beneficial species. Furthermore, herbal medicine components can also selectively target pathogens without disrupting the overall gut microbial balance. Alkaloids, flavonoids, saponins, and phenolic acids found in various herbal medicine formulations display antibacterial activity against specific pathogenic bacteria, such as *Escherichia coli*, *Staphylococcus aureus*, and *Helicobacter pylori* (Song et al. 2021; Bouchelaghem 2022; Morales-Figueroa et al. 2022). Furthermore, berberine, a bioactive alkaloid found in herbs, such as *Coptis chinensis* (Huang Lian), inhibits pathogens, such as *Escherichia coli* and *Salmonella typhimurium*, while enhancing the abundance of health-promoting bacteria, such as *Akkermansia muciniphila* (Kong et al. 2012; Dong et al. 2021). Notably, *Scutellaria baicalensis* (Huang Qin) contains flavonoids with potent antibacterial and anti-inflammatory effects, contributing to a more balanced gut microbiota (Cui et al. 2021). Herbal medicine is beneficial for maintaining a healthy gut microbiota composition and preventing dysbiosis-associated disorders by selectively suppressing the growth of harmful bacteria.

### Immunomodulation

The gut microbiota plays an essential role in modulating host immunity, and herbal medicine has been demonstrated to influence the composition and activity of the gut microbiota, subsequently impacting the host immune system. By enhancing the production of antimicrobial peptides and regulating inflammatory cytokines, herbal medicines can maintain the integrity of the gut barrier and prevent pathogen invasion (Liu et al. 2020a; Xia et al. 2022b). In addition, herbal medicine has been demonstrated to enhance the production of SCFAs, the primary metabolic products of gut microbiota fermentation, which have immunomodulatory effects by activating G-protein-coupled receptors and inhibiting histone deacetylases (Feng et al. 2022). Moreover, herbal medicine can also regulate the secretion of cytokines and chemokines, such as IL-10, IL-12, and transforming growth factor-beta (TGF-β), modulating both innate and adaptive immune responses (Fan et al. 2022; Lan et al. 2022; Li et al. 2023b). For instance, ginsenosides, the primary active compounds of *Panax ginseng* (Ren Shen), have been shown to regulate the expression of Toll-like receptors and other immune-related genes, leading to improved gut barrier function and microbial balance (Liang et al. 2021; Zhou et al. 2021).

One mechanism by which herbal medicine and the gut microbiota modulate immunity is by regulating the balance between different subsets of T cells. For example, certain herbal medicine-derived compounds enhance the proliferation of regulatory T cells (Tregs), which is pivotal for maintaining immunological tolerance and preventing autoimmune diseases. Concurrently, these compounds may also suppress proinflammatory Th17 cells, reducing inflammatory responses that could lead to tissue damage (Ang et al. 2020; Alexander et al. 2022; Ling et al. 2022). Moreover, the gut microbiota can modulate the activity of dendritic cells (DCs), which are essential for antigen presentation and activation of T cells. By influencing DC maturation and cytokine secretion profiles, herbal medicine compounds can shift the immune response toward either a more tolerogenic state or a heightened state of alert against pathogens, depending on the context and requirements of the host (Li et al. 2015; Mirza et al. 2018). Short-chain fatty acids (SCFAs), primarily acetate, propionate, and butyrate, are fermentation byproducts of dietary fibers generated by gut bacteria. SCFAs have been shown to exert multiple beneficial effects on immune homeostasis, including the suppression of inflammatory reactions and enhancement of mucosal barrier function. Many herbs used in TCM are rich in polysaccharides, which serve as prebiotics that promote the growth of SCFA-producing bacteria, thus fostering an anti-inflammatory environment (Tang et al. 2019). Another aspect of the gut microbiota–immune system interaction involves the gut–liver axis. Certain herbal medicines influence the influences gut microbiota composition and metabolic output, which in turn affects the immune-related functions of the liver. This cross-talk is crucial in conditions such as liver cirrhosis and hepatocellular carcinoma, where modulation of immune responses might be therapeutic.

### Gut barrier function and bile acid metabolism

Gut barrier function is crucial for maintaining gut homeostasis and bacteria and preventing bacteria and their metabolites from entering systemic circulation. Herbs rich in dietary fibers and polysaccharides promote the growth of beneficial bacteria such as *Lactobacillus* and *Bifidobacterium* species. These beneficial microbes can outcompete pathogenic bacteria, enhance gut mucosal immunity, and contribute to the production of short-chain fatty acids (SCFAs), which strengthen the gut barrier (Cheng et al. 2023; Wang et al. 2023a). Herbal medicine can enhance gut barrier function by upregulating the expression of tight junction proteins, such as occludin and claudins, and downregulating the levels of proinflammatory cytokines that disrupt the gut barrier, such as TNF-α and IL-1β (Yu et al. 2020; Liu et al. 2022a). These proteins are critical components of the tight junctions that seal the space between epithelial cells, preventing the leakage of luminal contents into the underlying tissue. Herbal medicine also appears to influence mucin production, which results in the formation of a protective layer on the epithelial surface, further enhancing barrier function. Herbs such as *Astragalus membranaceus* and Licorice roots have constituents that upregulate mucin gene expression, thereby contributing to the fortification of the mucosal barrier (Qiao et al. 2022). Furthermore, inflammation can compromise tight junction integrity. Herbal medicines possess anti-inflammatory properties and can reduce the levels of proinflammatory cytokines, such as TNF-α, IL-6, and IL-1β, which are known to disrupt gut barrier function. By downregulating these inflammatory mediators, herbal medicine contributes to maintaining tight junction integrity and preventing barrier dysfunction (Dey 2020). In conditions such as IBD, where the gut barrier is compromised, herbal medicine formulations have been shown to restore microbial balance, reduce inflammation, and improve tight junction protein expression, leading to enhanced barrier integrity and reduced disease severity.

In addition, bile acids are important signaling molecules that influence the composition of the gut microbiota and host metabolism. Herbal medicine has been shown to influence bile acid metabolism, thereby impacting physiological activities. The liver synthesizes primary bile acids, which can be modified by the gut microbiota into secondary bile acids. Herbal medicines such as Chai Hu (*Bupleurum chinense*) and Yu Jin (*Curcuma aromatica*) have been shown to regulate enzymes involved in bile acid synthesis, such as cholesterol 7 alpha-hydroxylase (CYP7A1), the rate-limiting enzyme in bile acid biosynthesis from cholesterol. By modulating the activity of such enzymes, herbal medicine can alter the composition and pool size of bile acids (Li et al. 2021b, 2022; Cheng et al. 2022). In addition, the gut microbiota plays an essential role in converting primary bile acids into secondary bile acids. Some herbal medicine compounds have antimicrobial properties that can selectively inhibit or promote the growth of specific bacterial species, thus influencing the bile acid transformation process. Other herbal medicine ingredients act as prebiotics, enhancing the growth of beneficial bacteria that contribute to a healthy bile acid profile. (Xiao et al. 2020). Furthermore, bile acids exert their effects through interactions with nuclear receptors, such as the farnesoid X receptor (FXR), and membrane-bound receptors, such as the G protein-coupled bile acid receptor (GPBAR1 or TGR5). Herbal medicines have been found to interact with these receptors, affecting glucose metabolism, lipid homeostasis, and energy balance. For example, certain herbal medicine-derived compounds can activate the FXR, leading to altered expression of genes involved in bile acid homeostasis and energy metabolism. Through the modulation of bile acid metabolism and receptor activation, herbal medicines can potentially affect several physiological processes. These include reducing cholesterol levels, improving glycemic control, and promoting energy expenditure (Hua et al. 2021; Zhang et al. 2022c). Additionally, because bile acids are involved in regulating inflammation and immune responses, herbal medicine-mediated manipulation of bile acid signaling pathways can have implications for treating inflammatory diseases.

### Other mechanisms

In addition to the mechanisms mentioned above, additional molecular pathways through which herbal medicine impacts the gut microbiota include the modulation of xenobiotic metabolism, the regulation of gut-derived hormone secretion, and the influence of bacterial metabolites, such as indole, trimethylamine N-oxide (TMAO), and secondary bile acids (Wang et al. 2021a; Zou et al. 2022). These multifaceted effects of herbal medicine on the gut microbiota further contribute to its therapeutic potential for various disorders.

In summary, herbal medicine offers numerous benefits for gut health through its ability to modulate the gut microbiota via diverse molecular mechanisms. However, further research is needed to elucidate the complex interactions among herbal medicine, the gut microbiota, and host physiology, paving the way for new therapeutic applications of herbal medicine in maintaining gut health and preventing disease.

### Herbal medicine: potential treatment for NDs through gut microbiota modulation

Recent studies have highlighted the potential of herbal medicine in the treatment of NDs by modulating the gut microbiota and influencing the gut–brain axis (Zhang et al. 2022a). In addition, research has demonstrated that herbal medicine can ameliorate cognitive decline, reduce neuroinflammation, and slow the progression of NDs by regulating the gut microbiota composition. For instance, a recent study revealed that treatment with *Ginkgo biloba* extract EGb 761 improved cognitive function in AD mice by restoring the balance of the gut microbiota and reducing neuroinflammation (Lautenschlager et al. 2012; Savaskan et al. 2018). Furthermore, another study revealed that berberine, a bioactive alkaloid derived from Coptis chinensis (Huang Lian), alleviated PD symptoms in a mouse model by modulating the gut microbiota and suppressing inflammation (Habtemariam 2020; Qin et al. 2020). Several herbs and formulas have shown promising effects on various NDs by modulating the gut microbiota. The following list highlights some of these therapeutic approaches.

### Alzheimer’s disease

*Huperzia serrata* (huperzine A): This herb has been demonstrated to inhibit acetylcholinesterase activity and reduce β-amyloid plaques in the brain while also improving cognitive function in AD patients. Its therapeutic effect may be associated with its impact on the gut microbiota (Friedli and Inestrosa 2021).

*Polygala tenuifolia* (senegenin): This herb has exhibited neuroprotective effects and has been reported to modulate the gut microbiota in AD mouse models (Xiong et al. 2022).

Formula: Liuwei Dihuang Wan is a commonly used formula that aims to nourish yin and strengthen the kidneys, thereby improving memory and cognitive function. Recent studies suggest that this formula may also influence the composition of the gut microbiota (Wang et al. 2022c).

### Parkinson’s disease

*Uncaria rhynchophylla* (Gou Teng, alkaloids, terpenoids and flavonoids): This herb possesses neuroprotective properties and has been shown to impact the composition of the gut microbiota in PD rodent models (Lan et al. 2018).

*Cistanche deserticola* (Rou Cong Rong, phenylethanol glycosides, iridoids, polysaccharides and volatile components): This herb is known to improve dopamine levels and herb has been found to alleviate some PD symptoms by altering the composition of the gut microbiota (Gao et al. 2021).

Formula: Zhenwu Tang is often prescribed for PD patients, aiming to warm and tonify yang while expelling dampness. Its therapeutic effects are potentially associated with gut microbiota regulation (Li 2020).

### Multiple sclerosis

*Scutellaria baicalensis* (Huang Qin, baicalin and baicalein): This herb, known for its anti-inflammatory and neuroprotective effects, has been reported to improve MS symptoms by modulating the gut microbiota (Wang et al. 2022b).

*Astragalus membranaceus* (Huang Qi, polysaccharides, flavonoids, and saponins): This herb helps boost the immune system and may alleviate MS symptoms by influencing the gut microbiota composition (Peng et al. 2023).

Formula: Bu Zhong Yi Qi Tang is commonly used for MS patients, aiming to tonify qi and strengthen the spleen. Its therapeutic effects have been associated with gut microbiota modulation (Lee et al. 2018).

In summary, herbal medicine holds substantial potential for treating NDs via gut microbiota modulation. However, further research is needed to elucidate the underlying mechanisms involved and identify optimal treatment strategies that harness the full potential of these ancient remedies.

## Conclusion and future perspectives

The exploration of herbal medicine about the gut microbiota has opened new vistas in the management of NDs. This review revealed that the gut–brain axis serves as a critical communication pathway through which the gut microbiota can significantly influence brain health and disease progression. The therapeutic potential of herbal medicine for NDs, such as AD, PD, HD, ALS, and MS is becoming increasingly apparent through emerging research. Notably, herbal medicine compounds have been shown to exhibit neuroprotective effects by modulating the gut microbiota composition, reducing systemic inflammation, and enhancing gut barrier integrity. This interplay between herbal medicine and the gut microbiota appears to extend to NDs, where modulation of the gut microbiota could mitigate neuroinflammation and oxidative stress—two central pathological features of NDs. The use of herbal medicine formulations, such as *Ginkgo biloba*, *Ginseng*, and *Polygala tenuifolia*, has demonstrated promising effects in preclinical studies. These herbs contain active metabolites that, once biotransformed by the gut microbiota, can cross the blood–brain barrier and exert therapeutic effects directly within the central nervous system.

Despite these encouraging findings, several challenges persist. Standardization of herbal preparations, understanding of individual phytochemical interactions with diverse gut microbiota constituents, and translation of preclinical results into clinical efficacy remain significant hurdles. Moreover, unraveling the specific components responsible for the beneficial effects is inherently complex due to the multifaceted nature of herbal medicine. The interindividual variability in the gut microbiota also poses a challenge for creating generalized treatments and underscores the need for personalized approaches. Advancing and integrating high-throughput sequencing technologies and metabolomics will enhance our understanding of the gut microbiota in healthy individuals and patients with NDs and elucidate the complex mechanisms underlying the action of herbal medicine on the gut–brain axis. The potential symbiotic relationship between prebiotic and probiotic treatments derived from or inspired by herbal medicine should be explored further to manipulate the gut microbiota beneficially. There is an opportunity to develop novel therapeutic agents targeting the gut–brain axis that can slow or halt the progression of NDs.

In conclusion, the intricate relationships among herbal medicine, the gut microbiota, and NDs hold immense untapped potential for innovative treatment strategies. As we advance our scientific and clinical understanding in this field, the integration of herbal medicine into mainstream ND management offers a hopeful avenue for millions affected by these debilitating conditions.
